# A first insight into the Polish Bochnia Salt Mine metagenome

**DOI:** 10.1007/s11356-023-25770-7

**Published:** 2023-02-13

**Authors:** Jakub Lach, Klaudyna Królikowska, Monika Baranowska, Magdalena Krupińska, Dominik Strapagiel, Agnieszka Matera-Witkiewicz, Paweł Stączek

**Affiliations:** 1 Department of Molecular Microbiology, Faculty of Biology and Environmental Protection, University of Lodz, Lodz, Poland; 2 Biobank Lab, Department of Oncobiology and Epigenetics, Faculty of Biology and Environmental Protection, University of Lodz, Lodz, Poland; 3 Department of Invertebrate Zoology and Hydrobiology, Faculty of Biology and Environmental Protection, University of Lodz, Lodz, Poland; 4 Screening of Biological Activity Assays and Collection of Biological Material Laboratory, Faculty of Pharmacy, Wroclaw Medical University Biobank, Wroclaw Medical University, Wroclaw, Poland

**Keywords:** Biodiversity, Biosynthetic gene cluster, Metagenomics, Salt mine

## Abstract

The Bochnia Salt Mine is one of the oldest mines in Europe. It was established in the thirteenth century, and actively operated until 1990. The mine has been placed on the UNESCO World Heritage List. Previous research describing Polish salt mines has been focused on bioaerosol characteristics and the identification of microorganisms potentially important for human health. The use of Polish salt mines as inhalation chambers for patients of health resorts has also been investigated. Nevertheless, the biodiversity of salt mines associated with biotechnological potential has not been well characterized. The present study paper examines the biodiversity of microorganisms in the Bochnia Salt Mine based on 16S rRNA gene and shotgun sequencing. Biodiversity studies revealed a significantly higher relative abundance of *Chlamydiae* at the first level of the mine (3.5%) compared to the other levels (< 0.1%). *Patescibacteria* microorganisms constituted a high percentage (21.6%) in the sample from site RA6. Shotgun sequencing identified 16 unique metagenome-assembled genomes (MAGs). Although one was identified as *Halobacterium bonnevillei*, the others have not yet been assigned to any species; it is possible that these species may be undescribed. Preliminary analyses of the biotechnological and pharmaceutical potential of microorganisms inhabiting the mine were also performed, and the biosynthetic gene cluster (BGC) profiles and antimicrobial peptide (AMP) coding genes in individual samples were characterized. Hundreds of BGCs and dozens of AMP coding genes were identified in metagenomes. Our findings indicate that Polish salt mines are promising sites for further research aimed at identifying microorganisms that are producers of potentially important substances with biotechnological and pharmaceutical applications.

**Supplementary Information:**

The online version contains supplementary material available at 10.1007/s11356-023-25770-7.

## Introduction

Halophiles are a highly diverse class of extremophilic organisms with high salinity requirements which comprises entities from all three domains of life (Ma et al. 2010). Due to their production of active biomolecules for biotechnological and pharmaceutical applications, and their unique metabolism, low nutritional requirements and adaptability to harsh conditions, halophilic microorganisms are attractive candidates for use in biomedicine and industry. Indeed, in the era of multidrug-resistant bacteria, a growing incidence of cancer, and extreme environmental pollution, research on the development of new active compounds is urgently needed. A number of valuable compounds extracted from halophiles have found applications in biotechnology, cosmetics, and medicine, such as ectoine, biosurfactants, and antimicrobial agents such as streptomonomicin (Amoozegar et al. 2017; Corral et al. 2020; Desai et al. 2020; Gómez-Villegas et al. 2020; Lach et al. 2021; Singh and Singh 2017).

Hypersaline ecosystems and their habitats have been widely explored mainly because of their unique biodiversity, and studies are currently focussing on environments related to salt production, aquaculture, and food production (Delgado-García et al. 2018; Pal et al. 2020; Van Thuoc et al. 2020). Their findings have led to a better understanding of the ecology of recently discovered groups of microorganisms such as DPANN, a superphylum of Archaea living in salterns that is characterized by a smaller cell size than other archaea (Pal et al. 2020). Also, new strains of halophilic microorganisms have been isolated from food products. One example is the strain *Salinivibrio proteolyticus* M318 isolated from fermented shrimp paste, which can be used to produce polyhydroxyalkanoates and ectoines (Van Thuoc et al. 2020). Studies of saline soils, which serve as reservoirs for many unique halophile species, have been found to contain new strains of halophilic microorganisms belonging to *Halobacillus* spp., *Marinococcus* spp., and *Alkalibacillus* spp. (Delgado-García et al. 2018). Newer methods based on omics are increasingly being used to analyze the soil microbiome to obtain a better understanding of the rhizosphere microbiome; this may have important implications for agriculture (Mukhtar et al. 2019). However, there are still many hypersaline environments that have not been sufficiently studied.

One such unexplored environments is the salt mine located in Bochnia, Poland. It is one of the oldest, and longest operating, salt mines in Europe, established in the thirteenth century and actively exploited until 1990. Hence, Bochnia Salt Mine is a unique natural and historical treasure which has been placed on the UNESCO World Heritage List (UNESCO World Heritage Centr n.d.). It was first established in a fragment of the marine sediments of the Miocene salt-bearing formation. The salt deposit in the Bochnia area was formed as a result of tectonic enrichment of the initially thin salt rock layers. Its geological structure has been relatively well characterized in the literature (De Leeuw et al. 2010; Garlicki 2008; Maj 2017; Poborski 1952; Wagner et al. 2010). Currently, the historic mine consists of nine post-mining galleries reaching 350 m below the surface, and a significant part of the excavations is open to the public (Puławska et al. 2021). Since the cessation of salt mining activity, the Bochnia Salt Mine has mainly been focused on tourism, recreation, and health protection, with the number of visitors increasing from 1500 in 1990 to over 133,500 in 2011 following the adaptation of the mine for tourism activities and its recognition as a UNESCO World Heritage Site (Krupa and Dec 2014; Wiewiórka et al. 2009).

The microbiota of salt mines in Poland has been the subject of scientific interest during the last decade. However, due to the function of mines as inhalation chambers for patients of health resorts, most studies have focused on bioaerosols. Their findings indicate that the air in salt chambers is characterized by a reduced level of biological contamination, including pathogen contents (Gębarowska et al. 2018; Górny et al. 2020; Myszkowska et al. 2019). Studies on the origin and health significance of airborne dusts in the Bochnia Salt Mine have also failed to detect any potentially toxic substances in the air. In addition, the presence of salt particles and salty spray in the atmosphere of salt mine may also have a beneficial effect on health (Puławska et al. 2021). However, none of the previous studies attempted a detailed characterization of the microbial community of the environment. In the only study of Polish salt mines to use next generation sequencing (NGS) to date, Goraj et al. (2021) describe the structure of the microbial community inhabiting halite and its importance for carbon transformation. In contrast, no studies have examined the brines from seepages and outflows in salt mines, nor have they assessed the biotechnological and pharmaceutical potential of the microbiota of Polish salt mines.

It has been shown that new species of halophilic microorganisms can be found in salt mines. In 2016, a new species of halophilic microorganism *Halorhabdus rudnickae* sp*.* nov. was isolated from a brine sample from a borehole in the unexploited Barycz mining area near Bochnia (Albuquerque et al. 2016). Research conducted in other parts of the world has also shown that salt mines are environments where unique organisms can be found, such as *Halobellus captivus* sp. nov., *Halococcus salifodinae* sp. nov., and *Halorubrum trueperi* sp. nov. (Chen et al. 2020, 2017; Denner et al. 1994). Studies conducted on entire microbial communities, such as in the Karak Salt Mine, Pakistan, found them to be dominated by bacteria, which accounted for more than 65% of the community (mostly *Bacteroidetes* and *Proteobacteria*). Only less than 20% of the microbial population of this mine consisted of archaea (Cycil et al. 2020). In contrast, a study of the microbial biodiversity of the Kilroot Salt Mine in Carrickfergus, Northern Ireland, showed a higher abundance of haloarchaea in an NaCl stalactite than samples from the nearby brine pools and soils; in addition, the metagenomes from NaCl stalactite also contained more genes related to adaptation to extreme environments (Thompson et al. 2021).

The effect of halite entrapment on halophilic microbial community composition has also been analyzed. The results indicate that halite-trapped archaeal communities were resilient to entrapment durations of up to 21 weeks and that such a halite entrapment may be an effective survival strategy for haloarchaea (Huby et al. 2021). In addition, ancient microorganisms trapped in halite fluid inclusions were identified in the USA, China, and Australia (Chen et al. 2021; Vreeland et al. 2000). Moreover, 830-million-year-old cell forms in fluid inclusions were observed in halite from Browne Formation (Australia). If this observation is confirmed by further biological analysis, it will be possible to identify sequences of genomes of the oldest halophilic microorganisms discovered so far (Schreder-Gomes et al. 2022). Some of the isolated microbes also encoded potentially valuable enzymes, such as a novel ω-transaminase (Kelly et al. 2018).

Due to the scarcity of biodiversity studies of brines from salt mines and the lack of assessment of the biotechnological potential of halophilic microbial communities, the aim of this study was to estimate the biodiversity of the microbiome of the Bochnia Salt Mine and to assess its biological potential, with particular emphasis on the detection of biosynthetic gene clusters (BGCs) and genes encoding antimicrobial peptides (AMPs). The historical significance of the Bochnia Salt Mine and its isolation from other saline environments characterizing this particular microbiome can be considered as a valuable supplement to the knowledge of the halophiles inhabiting salt mines in Europe and elsewhere.

## Materials and methods

### Sample collection and processing

**Fig. 1:**
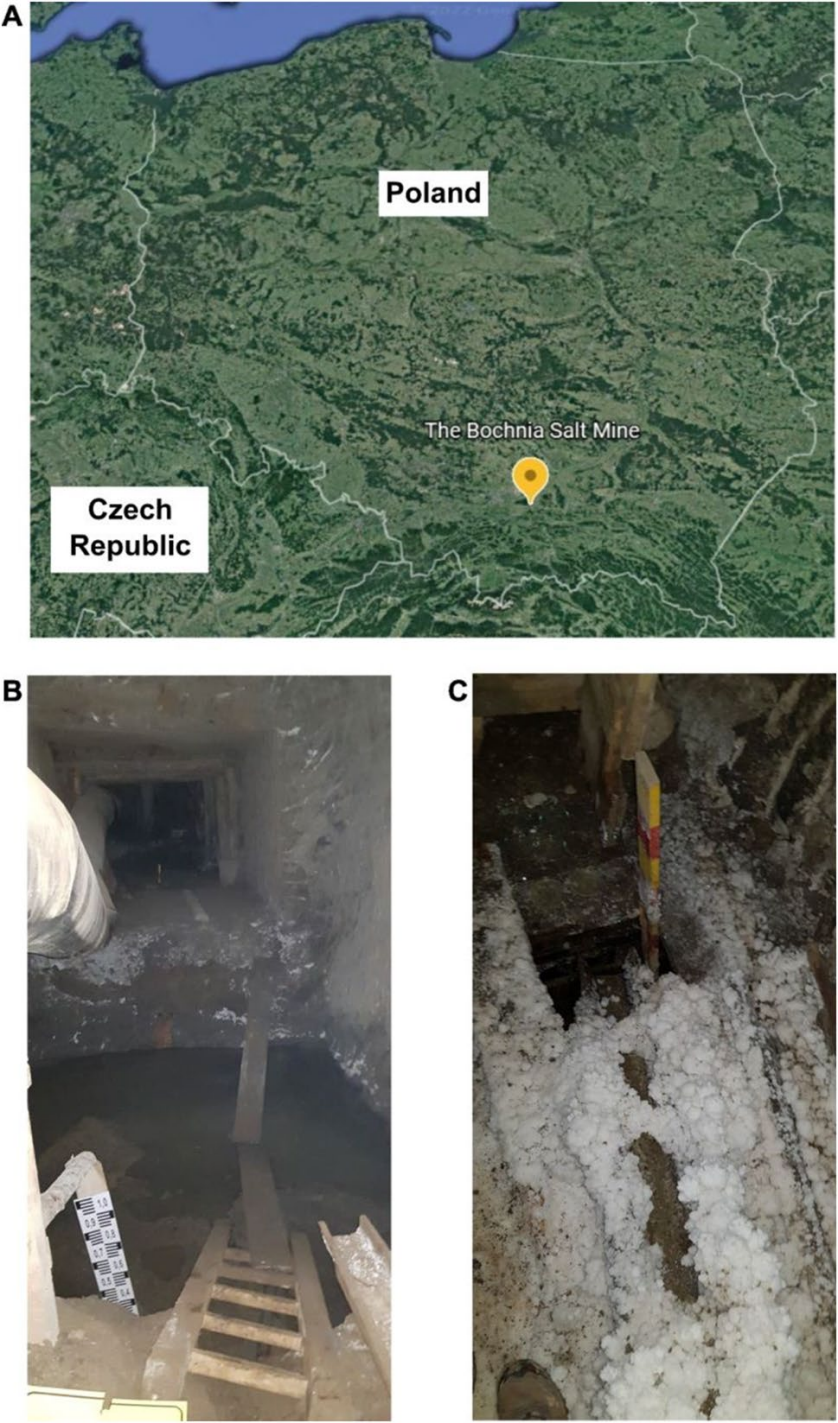
**A** The location of the Bochnia Salt Mine on the map of Poland. Examples of sampling sites: **B** RDB and **C** RA10

Samples were collected at the Bochnia Salt Mine located in the southern part of the Carpathian Foreland, near Kraków, Poland (49°58′09″N 20°25′03″E). Rock salt mining took place in the mine from the thirteenth century until 1990 (UNESCO World Heritage Centre n.d.). The inner tectonics of the Bochnia deposits reveal a unique accumulation of steep folds of large amplitude. Apart from the halite deposits, the mine contains layers of various rocks, including anhydrite, shaley marly claystone, zuber, and anhydritic claystone (Garlicki 2008). The current structure of the mine consists of nine post-mining galleries extending down to 350 m below the surface (Puławska et al. 2021). The sampling sites are presented in Fig. 1.

Brine samples collected from seven sampling sites in the Bochnia Salt Mine and analyzed by the Hydrogeochemical Laboratory of the Department of Hydrogeology and Engineering Geology at the AGH University of Science and Technology, Krakow, showed that the brines were characterized by similar salinity and pH throughout the mine (Interewicz 2018). The brines were nearly saturated with NaCl, with the salinity ranging from 31.3 to 33.3% and the pH was close to neutral, ranging from 6.6 to 7.4. More significant differences between sampling sites were observed in ion concentration levels. Brines with the highest mineralization level (based on Ca^2+^, Mg^2+^, and K^+^ ion concentrations) were collected from sampling sites POP27 and RA6. Samples from the first level of the mine were generally characterized by lower mineralization than those from the third and fourth levels. Brine collected from site POP27 (third level) contained the highest level of SO_4_^2−^ (111 g/l), with this value being over ten times higher than other samples (Interewicz 2018).

Twenty-four water samples were collected from brine wells at three levels of the mine in 2017 and 2019 and used for 16S rRNA gene sequencing. In 2017, 17 samples were collected: ten at level 1 of the mine (Danielowiec), four at level 3 (Wernier), and three at level 4 (August); in 2019, seven samples were collected: four at level 1 and three at level 4. In addition, one sample each was taken from levels 1, 2 (Sobieski), 3, and 6 (Sienkiewicz) for shotgun sequencing. The choice of sampling sites was related to the presence of brine reservoirs and flows in the mine, and the safety of access; due to the age of the mine and the state of its maintenance and ventilation, some levels and a significant number of locations were inaccessible for sampling.

The brine samples were collected into sterile 50-ml Falcon tubes and stored at 4 °C. To prepare the material for DNA isolation, 2 ml of saline was transferred into a new sterile Eppendorf tube and centrifuged for 10 min at 14 000 × *g*. After centrifugation, 1.8 ml brine was withdrawn and 1.8 ml of new brine was added. The centrifugation was then repeated. This procedure was repeated five times for each sample. Samples prepared in this way were then used for DNA isolation using the PowerSoil DNA Isolation Kit (Mo Bio Laboratories Inc., Carlsbad, CA, USA). The samples were eluted with nuclease-free water in a volume of 50 µl. DNA concentration was determined using the Qubit high-sensitivity (HS) assay kit (ThermoFisher, Waltham, USA).

### Sequencing and data availability

16S rRNA gene amplicon sequencing libraries were prepared according to the 16S Metagenomic Sequencing Library Preparation Illumina protocol (https://support.illumina.com/documents/documentation/chemistry_documentation/16s/16s-metagenomic-library-prep-guide-15044223-b.pdf). Shotgun sequencing libraries were prepared using the Vazyme TruePrep DNA Library Prep Kit V2 for Illumina (Nanjing, China). Throughout the DNA library preparation, negative controls without DNA were used to monitor for contamination. 16S rRNA metagenomic libraries were sequenced on an Illumina MiSeq with paired end reads. The libraries prepared for shotgun sequencing were sequenced on an Illumina NextSeq 500 with paired end reads.

All sequencing data are publicly available from the National Institutes of Health under BioProject accession PRJNA848445.

### Data analysis

At the first stage of the analysis, the quality of reads from both the 16S rRNA gene and shotgun sequencing protocols were checked using FastQC (Andrews 2022). In the next step, adaptors and low-quality sequences were removed from the reads with Trim Galore! v. 0.6.4 with default parameters (Krueger 2022).

Further analysis of the 16S rRNA gene samples was performed with QIIME 2 2021.8 (Bolyen et al. 2019). DADA2 was used for denoising data and ASVs (amplicon sequence variants) table generation. Both forward and reverse reads were trimmed at nucleotide 20 on the 5′ side, and at nucleotides 245 and 230, respectively, on the 3′ side. Alpha and beta diversity metrics were generated with the core-metrics-phylogenetic plugin with a sampling depth of 11,542, i.e. the number of reads that all samples were downsampled by to normalize them and reduce bias associated with differences in sequencing depths between individual samples.

Alpha diversity comparisons were performed between groups by the QIIME 2 “diversity alpha-group-significance” plugin with the Kruskal–Wallis test for both “all groups” and “pairwise” tests (Kruskal and Wallis 1952). For beta diversity comparisons, the QIIME 2 “diversity beta-group-significance” plugin with the PERMANOVA test was used (Anderson 2001). Taxonomic classification was performed using the feature-classifier classify-consensus-vsearch plugin based on pre-formatted SILVA reference sequence and taxonomy files from QIIME 2 data resources. Sequences were also functionally annotated using PICRUSt2 v. 2.4.1 (Douglas et al. 2020). Analysis of composition of microbiomes (ANCOM) was used to identify features that differed in abundance between groups (Mandal et al. 2015). Before ANOCOM, the taxa present only in one sample were removed.

For shotgun sequencing data, de novo assembly was performed using MEGAHIT v.1.2.9 with a minimum length of 1000 bp of contigs obtained (Li et al. 2015). The quality of the assemblies was checked using metaQUAST (Mikheenko et al. 2016). The contigs were binned to the metagenome-assembled genomes (MAGs) with metaBAT2 v. 2:2.15 (Kang et al. 2019). For dereplication of MAGs, dRep v3.2.2 was used (Olm et al. 2017). MAGs with a minimum length 50,000 bp, contamination lower than 25%, and completeness higher than 75% were taken for further analysis. Metagenome gene annotations were performed using DFAST version 1.2.14 (Tanizawa et al. 2018). BGC prediction was performed with antiSMASH v.6.0.1 (Blin et al. 2021) and deepBGC v. 0.1.27 (Hannigan et al. 2019). Taxonomic annotation of MAGs was performed with gtdbtk version 1.5.1 (Chaumeil et al. 2020) and at the read level using Kaiju 1.8.2 (Menzel et al. 2016). Identification of AMP sequences was performed using marcel v. 0.3.1 on default parameters (Santos-Júnior et al. 2020). AMP sequences with haemolytic properties were verified and classified into functional types by using iAMP-2L platform (Xiao et al. 2013).

## Results

### Sequencing statistics

NGS was used across the V3-V4 region of the 16S rRNA gene to sequence 24 environmental samples. The mean number of ASVs per sample obtained after DADA2 was 72,050 ± 31,480, ranging from 22,568 to 143,267. From a total of 1,729,209 contigs remaining after quality control and removal of chimeras, a total of 3655 unique ASVs were obtained. These ASVs represented 46 phyla, 93 classes, 209 orders, 352 families, and 635 genera within the domains of bacteria and archaea.

### Sampling season comparison

**Fig. 2:**
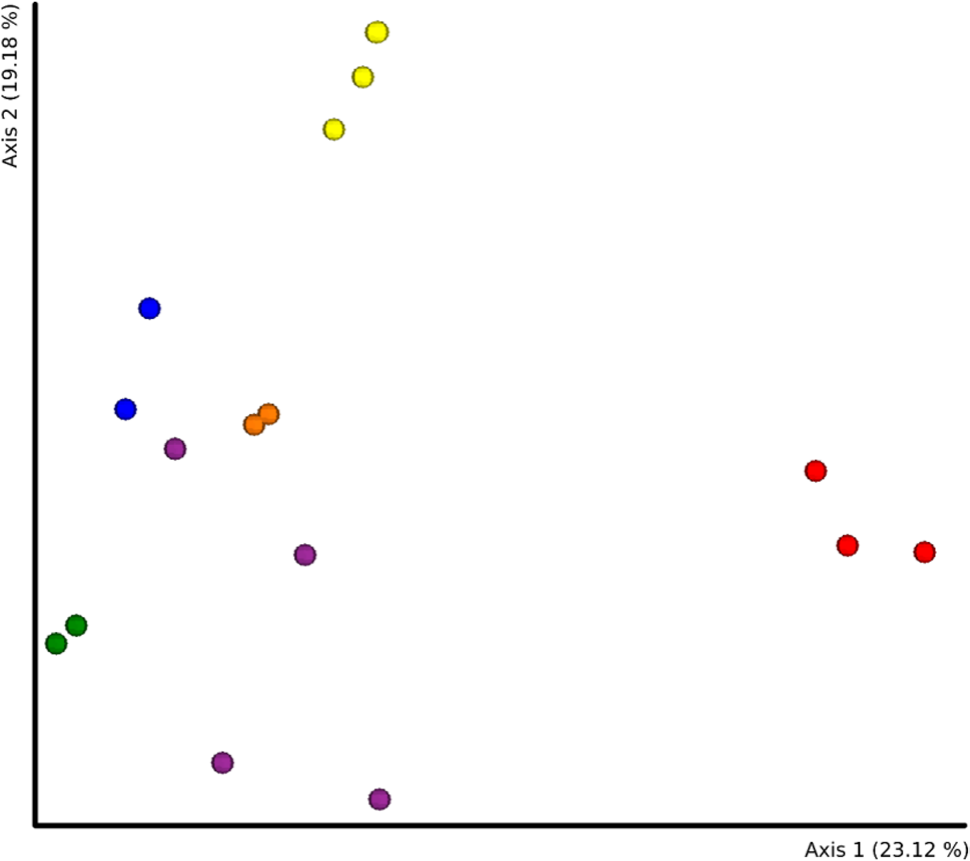
PCoA calculated on Bray–Curtis distance for samples collected in a single location in both seasons. Yellow—RDG, blue—RA6, orange—RD10, purple—RDB, green—RD13, red—RA10

To compare possible changes between sampling seasons, the results of 16S rRNA amplicon sequencing are presented for 17 samples that were selected from sites analyzed in both seasons. The Bray–Curtis distance was used for Principal Coordinate Analysis (PCoA); the results indicate that the samples were clustered based on sampling sites (Fig. 2). Only samples from the RDB site (purple) were not placed in a homogeneous cluster. However, clustering by season was not demonstrated. A comparison of Bray–Curtis distances showed no statistically significant difference between seasons (pseudo-*F* = 1.020224, *p* = 0.381). The difference between locations was globally significant (pseudo-*F* = 3.67932, *p* = 0.001). The pairwise tests identified significant differences between RDG and RA10 (pseudo-*F* = 9.047075, *p* = 0.043), RDG and RDB (pseudo-*F* = 3.361130, *p* = 0.023), and RDB and RD10 (pseudo-*F* = 3.245796, *p* = 0.029). No statistically significant differences were found between locations based on the Shannon index. The results suggest that the microbiome of each site was stable over time, allowing samples from both seasons to be used to compare the microbiome of the mine sampling sites in the further analyses.

### Sampling site and mine level comparison

**Fig. 3:**
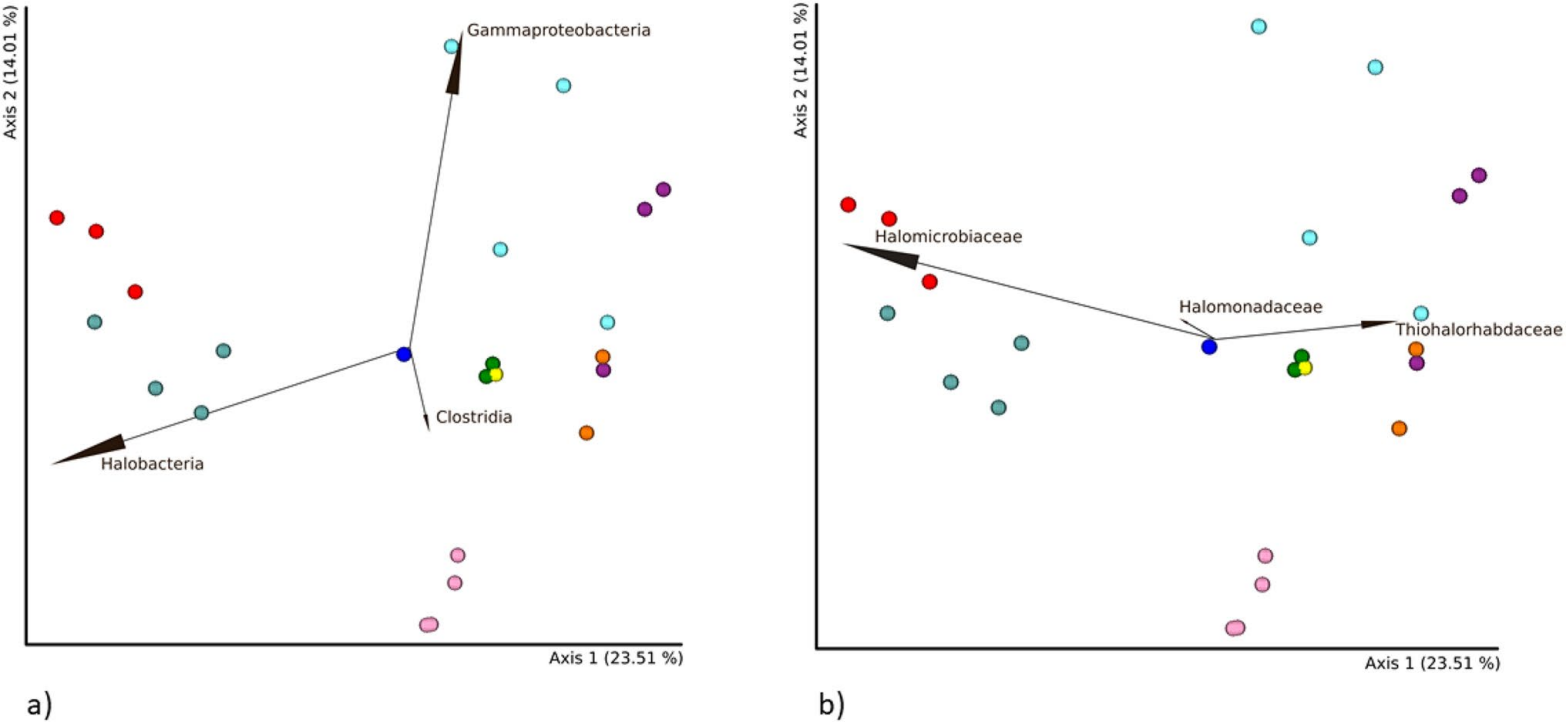
Biplot projecting taxonomic abundance at (**a**) class and (**b**) family level onto a principal component matrix (PCM) generated based on the Bray–Curtis distance

The microbiomes of all 24 brine samples from individual sampling sites were compared. PCoA based on Bray–Curtis distances confirmed partial clustering of samples depending on the sampling site (Figure S1). Separate clusters were formed by samples from the RDG and RD10/Wernier sites. The results also indicate that samples from other sampling sites formed one common, mixed cluster. No statistically significant differences were found between individual sites or mine levels based on the Shannon index. However, analysis based on the Bray–Curtis distance revealed a statistically significant difference at the global level (pseudo-*F* = 2.707996, *p* = 0.001), pairwise differences between Level 1 and Level 3 (pseudo-*F* = 3.633673, *p* = 0.002) and between Level 1 and Level 4 (pseudo-*F* = 2.268532, *p* = 0.015). Figure 3 shows the biplot projecting the taxon abundance onto the principal component matrix generated from the Bray–Curtis distance. The taxa most strongly influencing the location of samples on the PCoA were Halobacteria, Gammaproteobacteria, and Clostridia, at the class level, and *Halomicrobiaceae*, *Thiohalorhabdaceae*, and *Halomonadaceae* at the family level*.*

ANCOM analysis showed that microorganisms belonging to the Chlamydiae class of the phylum Verrucomicrobiota were significantly more abundant in the samples from level 1 of the mine than other levels, where the members of this class were almost absent. The mean content of the Chlamydiae sequence was 3.5% in samples from level 1 and 0.04% for level 4; the sequence was absent from level 3. The highest occurrence of this taxon was observed in samples from the RD13 site, where Chlamydiae accounted for 10.07% of all ASVs in the 2017 season, and 12.28% in the 2019 season. Samples from the RDB and RDG sites on level 1, like the other mine levels, had low levels of ASVs belonging to the Chlamydiae (< 1%). Within this class, most sequences belonged to the *Simkaniaceae*, but sequences belonging to families such as *Omnitrophaceae* or *Puniceicoccaceae* were also found at lower frequencies. Samples from locations RA3, RD13, and RDB had an increased proportion of *Desulfobacteraceae* representatives in the microbiota, and samples from RD13 and RDB sites additionally contained more *Thioalkalispiraceae* than other locations. The latter are microorganisms that require sulphur for growth, and their presence may be related to the structure of the mine rocks, especially the presence of anhydrite which may be a source of this element.

Samples from level 1 and level 4 of the mine contained mainly bacterial ASVs (74.8% ± 12.2% and 61.5% ± 14.8%, respectively), while samples from level 3 had a more balanced microbiome, consisting mainly of archaea (52.2% ± 7%) and bacteria (47.4% ± 7.1%). The two predominant phyla were Proteobacteria and Halobacterota. The samples from the first level of the mine contained the most Proteobacteria (31.7% ± 18.7%); the main contributors were the two samples from the RDB site, which contained 81.9% and 62.9% of Proteobacteria, respectively. In contrast, the samples from levels 3 and 4 contained 22.5% ± 10.1% and 29.7% ± 10.7% of Proteobacteria, respectively. The mean prevalence of Halobacterota was 19.5% ± 10.4% for samples from level 1, 42.3% ± 6.2% for samples from level 3, and 30.0% ± 13.8% for samples from level 4.

It is also worth noting that all samples contained bacteria belonging to the phylum Desulfobacterota, capable of metabolizing sulphur compounds. This taxon was most abundant in the first mine level, and particularly the sample from the RDB sampling site (31.5%), where the mean value for this level was 9.7%. Most of the Desulfobacterota in the RDB site were found to belong to the genera *Desulfovermiculus* and MSBL7; these were commonly accompanied by the genus *Bradymonadaceae* in other samples. The phyla Nanohaloarchaeota and Firmicutes were also present in all samples, both at an average level of 6.4%.

Samples from the RDG site were characterized by a high percentage of Halanaerobiaeota: its mean prevalence was 18.7%, compared to 3.7% for all samples. The main genera in the Halanaerobiaeota were *Halanaerobium* and *Halanaerobacter*, which were responsible for most of the ASVs assigned to this phylum. One interesting case was presented by the sample from the RA6 site, where an important element of the microbiome was the phylum Patescibacteria, comprising microorganisms with highly reduced genomes (< 1Mbp). In this sample, Patescibacteria accounted for 21.6% of the total ASVs, while the mean value for all samples was 3.5%.

**Fig. 4:**
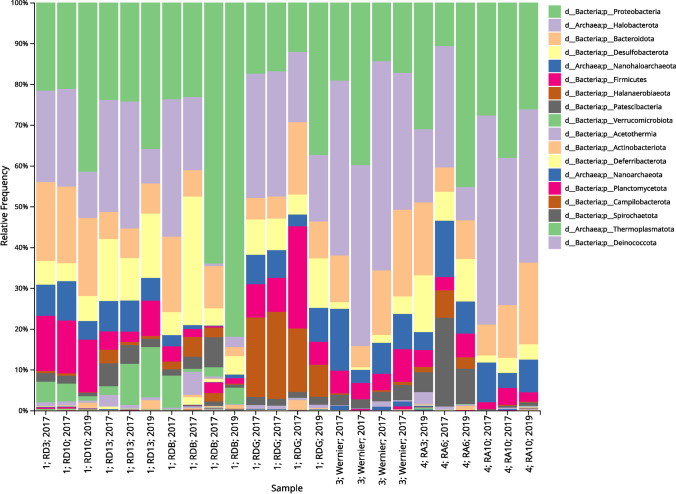
Taxonomic composition of samples at phylum level. Samples are sorted by salt mine level and sampling sites

In the RA6 sample, the dominant species within the Patescibacteria was *Candidatus Falkowbacteria*, which accounted for 18.6% of the microbial composition of the site. The taxonomic composition of the samples is shown in Fig. 4.

In total, 46 phyla were identified throughout all the 24 analyzed samples. Nine of these phyla were represented in all samples, making up the main part of their microbiome: Halobacterota, Proteobacteria, Bacteroidota, Desulfobacterota, Nanohaloarchaeota, Firmicutes, Patescibacteria, Halanaerobiaeota, and Acetothermia. Only two of these common phyla: Nanohaloarchaeota and Halobacterota, belonged to the Archaea. Moreover, the presence of Patescibacteria in all samples may be of interest since they are microorganisms with strongly reduced genomes, which is associated with limited possibilities of adaptation to stress conditions (Tian et al. 2020).

The taxa present in all samples comprised 14 of 96 classes, 20 of 209 orders, 22 of 352 families, and 26 of 635 genera. The genera found in all samples included *Natronomonas*, *Nanosalinaceae*, *Thiohalorhabdus*, *Salinisphaera*, *Halofilum*, *Haloplanus*, *Halobacterium*, *Halolamine*, *Desulfovermiculus*, *Bradymonadaceae*, *Alcanivorax*, *Halodesulfurarchaeum*, *Acetothermiia*, *Abscondimielaslaas*, *Pseudohlaxla*, and *Abscondimielaslaas*. The other genera were non-cultivable bacteria (designated unculturable in the SILVA database) belonging to the families *Balneolaceae*, *Halomonadaceae*, *Balneolaceae*, *Halomicrobiaceae*, and *Micavibrionaceae* or to the orders and classes (lowest level designated) Thermovenabulales, Chitinophagales, and Bacteroidia. At genus level, the most common taxa also belonged to the Bacteria (19 taxa) and only a small part were representatives of the Archaea (seven taxa); this is interesting as the very high salinity of the brine should favour the presence of extreme halophiles from the *Archaea* domain. The most representative phylum was *Proteobacteria*, which included 10 of the designated 26 genera. The presence of numerous taxa with sulphur-based metabolism may be associated with the occurrence of anhydrite in the mine (Garlicki 2008).

### Metabolic pathway analysis

The ASVs were annotated with BioCyc ID metabolic pathways using the PICRUSt2 tool. PCoA based on Bray–Curtis distance was used to cluster samples in three main groups, as previously with the taxa: one group contained samples from the Wernier and RA10 sites; a second group from the RDG site, with one sample from the 2019 season being an outlier, unlike the analysis at the ASVs level; and a third group that included the remaining samples, although in this case a clear clustering pattern was observed (Figure S2).

**Fig. 5:**
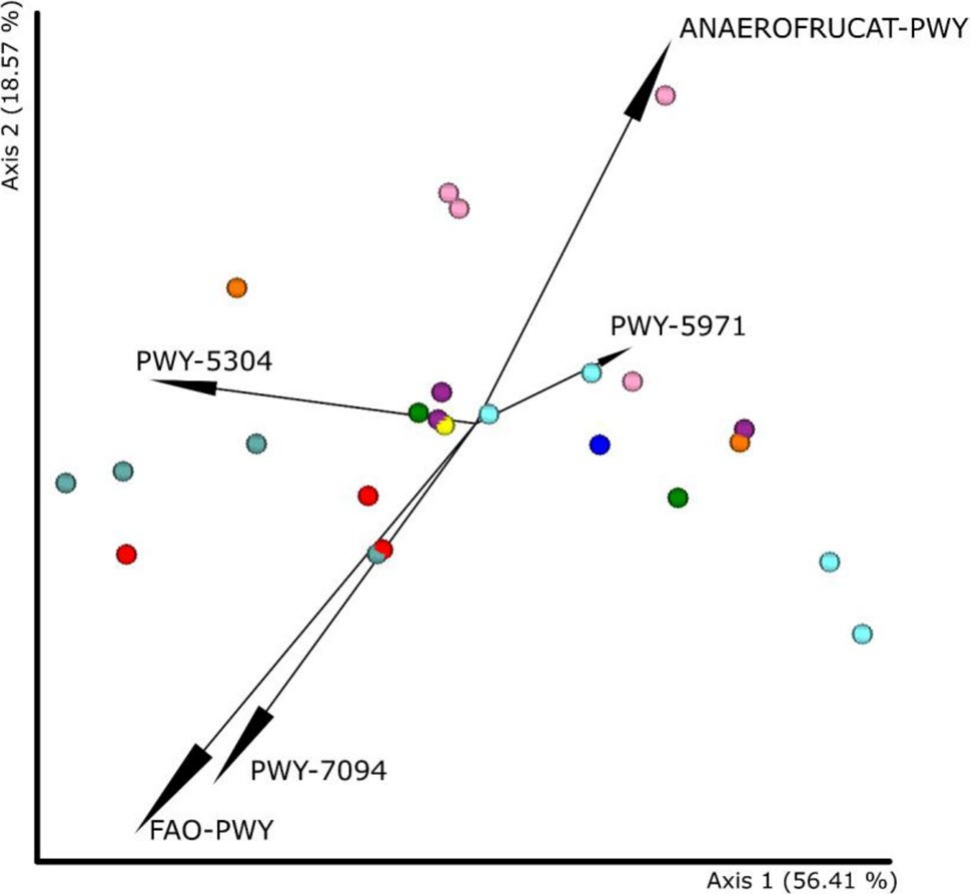
Biplot projecting metabolic pathway abundance onto a principal component matrix (PCM) generated from the Bray–Curtis distance. Red—RA10 (level 4), cadet blue—Wernier (level 3), light blue—RDB (level 1), purple—RD13 (level 1), dark blue—RA3 (level 4), green—RD10 (level 1), yellow—RD3 (level 1), orange—RA6 (level 4), pink—RDG (level 1)

Based on the biplot, five main pathways influencing the placement of samples on the PCoA were distinguished: PWY-5304 (sulphur oxidation superpathway (*Acidianus ambivalens*)), FAO-PWY (fatty acid β-oxidation I (generic)), PWY-7904 (tRNA- uridine 2-thiolation (thermophilic bacteria)), PWT-5971 (palmitate biosynthesis II (type II fatty acid synthase)), ANAEROFRUCAT-PWY (homolactic fermentation) (Fig. 5). The samples from the Wernier and RA10 sites demonstrated increased abundance of the PWY-5304, PWY-7904, and PWT-5971 pathways, while samples from the RDG site favoured the ANAEROFRUCAT-PWY. ANCOM analysis indicated that the samples from level 4 of the mine had higher participation of the P162-PWY, i.e. L-glutamate degradation V (via hydroxyglutarate), and PWY-5177 (glutaryl-CoA degradation) pathways than the other samples. The samples from the RA6 site demonstrated the highest share of the PWY-5177 (glutaryl-CoA degradation) pathway. In contrast, samples from RA3, RA6, RD13, RDG, RDB sites were characterized by increased levels of the P161-PWY, i.e. acetylene degradation (anaerobic) pathway. The only pathway present in samples from the RDB site was PWY-7046, i.e. the 4-coumarate degradation (anaerobic) pathways. In addition, samples from sites RA10, RD10, RD3, and Wernier had lower levels of the PWY-6749 (CMP-legionaminate biosynthesis I) pathway than the others.

Core feature analysis showed that 305 of 415 (73.5%) of the identified metabolic pathways were common to all samples. At least half of the samples contained 364 of the 415 lanes (87.7%). Based on the Shannon index, a statistically significant difference was demonstrated between mine level 1 and mine level 3 (KW global test result *p* = 0.007 and *q* = 0.008 for this pair), with samples from level 3 showing significantly lower path diversity than samples from level 1. This observation was also confirmed by the Bray–Curtis distance (*p* = 0.003; *q* = 0.003). This relationship was also evident in PCoA.

### Shotgun metagenomics analysis

Four samples were included in the analysis: W1 from the first level (Danielowiec), WSO4 from the second level (Sobieski), POP27 from the third level (Wernier), and W81 from the sixth level (Sienkiewicz). The total length of the metagenomes ranged from 25,265,906 to 71,844,617 bp. Each was composed of between 12,477 and 25,733 contigs longer than 1000 bp. The range of GC content was 52.0–61.7%. The number of CDS ranged from 14,004 to 49,587. The statistics concerning de novo assembly and binning are presented in Table S1, and the taxonomic assignment of reads at the phylum level is presented in Table 1.

**Table 1:** Taxonomic composition of metagenomic samples at the phylum level. The category “Others” contains reads assigned to viruses and phyla with an abundance lower than 0.1% in each of the samples. Unassigned reads were excluded from taxa abundance calculation

Sample name	W1 (level 1) (% of reads)	WSO4 (level 2) (% of reads)	POP27 (level 3) (% of reads)	W81 (level 6) (% of reads)
Acidobacteria	0.20	1.07	1.49	1.12
Actinobacteria	2.89	0	0	0
Bacteroidetes	5.91	1.14	0.91	0.25
Balneolaeota	4.75	0.25	0.16	0
Chlamydiae	0.19	0	0	0
Chloroflexi	0.26	0	0	0
Cyanobacteria	0.35	0.12	0.17	0.27
Euryarchaeota	62.63	88.19	74.61	92.67
Firmicutes	3.22	0.68	0.77	0.68
Ignavibacteriae	0.10	0	0	0
Nitrospirae	0.12	0	0	0
Planctomycetes	0.25	0	0	0
Proteobacteria	12.40	4.83	17.34	1.79
Spirochaetes	0.15	0	0	0
Thaumarchaeota	0.10	0	0	0
Verrucomicrobia	0.12	0	0	0
Others	6.36	3.72	4.55	3.22

The results indicate the dominance of Euryarchaeota with its prevalence ranging from 62.63% in W1 to 92.67% in W81. The second most represented phylum was Proteobacteria (1.79%—W81, 17.34%—POP27). Moreover, in sample W1, where the greatest diversity of taxa was observed, a significant proportion of Actinobacteria (2.89%) was found. At the genus level, all samples were characterized by a microbiome composition typical of high-salinity environments. Almost all identified genera belonged to the *Halobacteria* family, with *Halorubrum* being the most abundant genus: it was the most abundant genus in POP27, WSO4, and W81 samples, with 12.1%, 14.5%, and 16.1%, respectively. In sample W1, the most common genus was *Natronomonas*, accounting for 4.7% of the microbial community, and *Halorubrum* was the second most common, with an abundance of 3.9%. *Natronomonas* was also present as an important part of the microbiomes of the other samples. Another important taxon was *Halobacterium*, which was detected in all samples, and accounted for 3.3 to 16.1%.

**Fig. 6:**
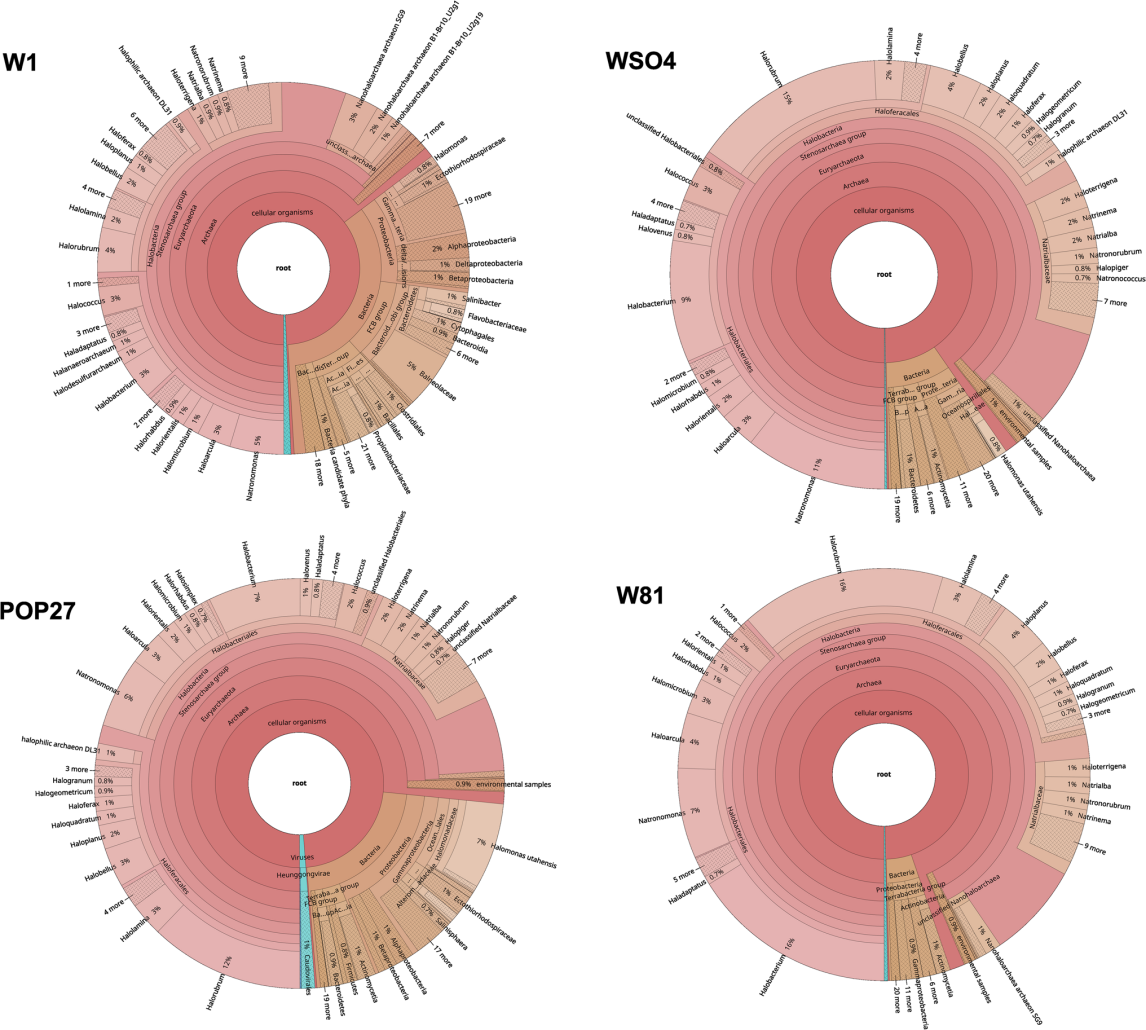
Taxonomic composition at the genus level based on shotgun sequencing data

Sample W1 was found to demonstrate a unique microbiome composition. First, more than 25% of the microbial community in this sample comprised bacteria, which was not the case in the other samples. It was also the only sample where bacteria other than *Proteobacteria*, belonging to the Bacteroidetes, Balneolaeota, Firmicutes, and Actinobacteria, accounted for more than 1% of the microorganisms. In addition, as mentioned earlier, the dominant type of archaea in this sample was *Natronomonas*, which was not the case with other samples. These findings emphasize the slightly different nature of the W1 sample compared to the first level of the mine, thus confirming the observations made through 16S rRNA analysis. Krona charts for each sample at the genus level are shown in Fig. 6.

The dRep tool was used for dereplication of the obtained MAGs. Ultimately, 16 unique MAGs with less than 25% contamination and more than 75% completeness were selected. The bin pairs WSO4.1-W81.7, WSO4.13-POP27.10, WSO4.4-POP27.1, WSO4.22-POP27.15, and WSO4.8-POP27.16 had paired Average Nucleotide Identity (ANI) values above 99%, which indicated that they belonged to the same species and probably the same strain. Therefore, only one genome from each pair was selected for further analysis as representative of the group. Finally, the following number of MAGs was identified in each sample: three in POP27, two in W1, seven in W81, and four in WSO4. The highest number of MAGs was identified in W81; this sample demonstrated the least diversity in the microbial community, which could have resulted in a better recovery of contigs belonging to the identified MAGs. The completeness of MAGs ranged from 76.2 to 98.28%, and contamination from 1.4 to 24.61%. The resulting MAGs were between 826,353 and 5,341,881 bp in length. Detailed statistics on the quality of the bins are presented in Table S2.

Taxa were assigned to bins using the GTDB-Tk tool, as shown in Table 2. Classification to species level was only possible in bin WSO4.4; the results indicated the species *Halobacterium bonnevillei*. In the remaining cases, the classification ended at the genus or family level, which may indicate that the bins belong to unknown species. Only four of the obtained MAGs were identified as bacterial: MAG POP27.15, assigned to the genus *Halospina*, W1.7 assigned to the order T1Sed10-126, and two MAGs, POP27.16 and WSO4.8, assigned to the family *Salinisphaeraceae*. The other MAGs identified as archaeal belonged to the Halobacteria and Nanosalinia classes. Interestingly, only two MAGs belonged to the Nanosalinia, and both were identified in the W81 sample.

**Table 2:** Taxonomic classification of unique bins

Bin name	Domain	Phylum	Class	Order	Family	Genus	Species
POP27.15	*Bacteria*	*Proteobacteria*	*Gammaproteobacteria*	*Pseudomonadales*	*Oleiphilaceae*	*Halospina*	*-*
POP27.16	*Bacteria*	*Proteobacteria*	*Gammaproteobacteria*	*Nevskiales*	*Salinisphaeraceae*	*-*	*-*
POP27.17	*Archaea*	*Halobacteriota*	*Halobacteria*	*Halobacteriales*	*Halobacteriaceae*	*-*	*-*
W1.4	*Archaea*	*Halobacteriota*	*Halobacteria*	*Halobacteriales*	*Haloarculaceae*	*Natronomonas*	*-*
W1.7	*Bacteria*	*T1Sed10-126*	*T1Sed10-126*	*T1Sed10-126*	*-*	*-*	*-*
W81.6	*Archaea*	*Nanohaloarchaeota*	*Nanosalinia*	*Nanosalinales*	*Nanosalinaceae*	*SG9*	*-*
W81.7	*Archaea*	*Halobacteriota*	*Halobacteria*	*Halobacteriales*	*Haloarculaceae*	*Natronomonas*	*-*
W81.9	*Archaea*	*Halobacteriota*	*Halobacteria*	*Halobacteriales*	*Halobacteriaceae*	*Halobacterium*	*-*
W81.14	*Archaea*	*Halobacteriota*	*Halobacteria*	*Halobacteriales*	*Haloferacaceae*	*Halorubrum*	*-*
W81.15	*Archaea*	*Nanohaloarchaeota*	*Nanosalinia*	*Halorubrum*	*Nanosalinaceae*	*M3-22*	*-*
W81.20	*Archaea*	*Halobacteriota*	*Halobacteria*	*Halobacteriales*	*Haloferacaceae*	*Haloplanus*	*-*
W81.21	*Archaea*	*Halobacteriota*	*Halobacteria*	*Halobacteriales*	*Haloarculaceae*	*Halomicrobium*	*-*
WSO4.1	*Archaea*	*Halobacteriota*	*Halobacteria*	*Halobacteriales*	*Haloferacaceae*	*Halolamina*	*-*
WSO4.4	*Archaea*	*Halobacteriota*	*Halobacteria*	*Halobacteriales*	*Halobacteriaceae*	*Halobacterium*	*Halobacterium bonnevillei PCN9*
WSO4.8	*Bacteria*	*Proteobacteria*	*Gammaproteobacteria*	*Nevskiales*	*Salinisphaeraceae*	*-*	*-*
WSO4.13	*Archaea*	*Halobacteriota*	*Halobacteria*	*Halobacteriales*	*Haloferacaceae*	*Halorubrum*	*-*

The BGCs were identified using two methods. The first one, antiSMASH, is based on the use of rules related to the construction of a cluster, and strongly focuses on the already existing BGCs. The second method, DeepBGC, is based on neural networks that give a better chance to identify previously undescribed BGCs, where functional domain analysis was applied. Metagenomes were also screened for the presence of AMP-encoding genes. The total number of BGCs detected in the metagenomes, the number of BGCs for each class, and the total number of detected AMPs are presented in Table 3.

**Table 3:** BGC and AMP statistics

Sample name	W1 (level 1)	WSO4 (level 2)	POP27 (level 3)	W81 (level 6)
AntiSMASH				
# of BGC	3	12	20	12
T3PKS	1	0	0	0
Arylpolyene	1	1	2	0
Betalactone	0	1	1	2
Ectoine	0	1	3	0
Lanthipeptide class II	0	0	0	2
RiPP-like	0	1	0	0
Siderophore	0	0	2	0
Terpene	1	8	11	8
Thiopeptide	0	0	1	0
DeepBGC				
# of BGC	136	753	856	558
Product class				
NRP	0	6	7	1
Other	0	19	17	18
Polyketide	16	86	109	52
RiPP	9	33	58	41
Saccharide	10	83	54	83
Terpene	4	24	20	15
Unclassified	99	516	604	350
Product activity				
Antibacterial	115	621	696	459
Antifungal	0	3	8	1
Cytotoxic	2	7	11	9
Inhibitor	1	10	9	5
Unclassified	18	115	140	85
AMPs				
# of AMPs	15	30	25	7
Haemolytic	12	20	14	6
Non-haemolytic	3	10	11	1

It can be seen that DeepBGC enabled the detection of significantly more potential BGCs than AntiSMASH. Both methods identified the highest number of potential BGCs in the sample from the third level of the mine, and the lowest number in the sample from level 1. It was also noteworthy that both methods detected BGCs for terpenes in all samples. In sample POP27, antiSMASH identified the complete operon ectABC, related to the biosynthesis of ectoine, and the operon iucABCD, encoding aerobactin biosynthetic proteins. A BGC related to thiopeptide synthesis was also identified. The metagenome of POP27 contained numerous BGCs organized around genes coding phytoene synthase, which may suggest that these clusters were related to the biosynthesis of carotenoids. In contrast, antiSMASH identified only three clusters in sample W1; these appear to be incomplete and, therefore, cannot be properly characterized. In sample W81, two BGCs associated with the production of class II lanthipeptides were identified. One of the clusters can most likely produce three lanthipeptides: MSVAIDNKAVIGG – RKKQFDAEFEDhbNDhbDDKDhaDDhbLGPAKLCIGDhaFDhbCVLNDhaRVVVP, VCERDASRLVNVAVRSRSQHSRFAVFNVSLSSSWLSIDTETNELLTSQTA – LDhbRQCRDhbNYCCNQDhbPIFDhbWLDhbV, LSIDTETNELLTSQTG – LDhbRQCLRAFRVRQELYRKPVCEALKHAVLADhbQQRFEINDVLRVDhaVAWKYALMDhbKWLYALRERF and the other is related to the production of four lanthipeptides: MSVAMSDDAGATVNKQAYAEEFNQSAPSTDHDKGEDTVGA – NCYLNDhaIVCDhbFDhbDhbGGQ, VPIIRTFIFLKTAATA – FERWGVRLCRARDhbPAEFLKLIIRLHLKNIAFKHDhaDhaFR, MAMAAPRRSGTSNESRSRSPAA – ADhbNDhaADhbGADhbMPDhbNCADhbVLAIDha, VSHHRHDEVPSA – LVEIAQQDhbAQQERERHVRECAAPGADVV. In the case of the WSO4 sample, as in POP27, the complete ectABC operon was identified. BGCs associated with the synthesis of a RiPP-like protein, most likely similar to Linocin M18, were also identified in this sample.

## Discussion

The salt mine in Bochnia is an excellent example of a hypersaline site, the analysis of which may contribute to a better understanding of the ecology and genomics of halophiles. Due to the great historical and natural value of the site, our exploration of the microbiota of the Bochnia Salt Mine is particularly important (UNESCO World Heritage Centr, n.d.). Our findings present a novel insight into the structure of the microbiota of the Bochnia Salt Mine using metagenomics, which provides a glimpse into the diversity, distribution, and functionality of the microbiome as well as its structure. As such, the present study is the first such investigation of the hitherto undescribed metagenomic community profiles of the halophile community from the Bochnia Salt Mine.

The observed differences in microbial community composition were probably not associated with the basic physicochemical properties of analyzed brines. This is due to the fact that brines in the Bochnia Salt Mine are characterized by similar salinity, pH, and levels of major ions. Each of the brines was almost saturated with NaCl and had a pH close to neutral. Therefore, it can be assumed that any recorded differences in the microbial composition were related to the location within the mine, as well as other factors such as surface water supply, or differences in brine composition at the level of trace elements.

The 16S rRNA analysis revealed significant differences between microbiome composition at different mine levels. A statistically significant difference in biodiversity was found based on Bray–Curtis distance between the first mine level and the third and fourth levels. In addition, a difference in the diversity of metabolic pathways assigned to ASVs was also observed between the first and third mine levels. These differences could be related to the mineralization of brine. The Ca^2+^, Mg^2+^, and K^+^ ion concentration was lower in brines from the first level than those from the third and fourth levels, which could have affected the microbial community structure. This observation was confirmed by PCoA analysis, which found the samples from the first level to be widely scattered. Samples from the other mine levels were more clustered.

ANCOM analysis showed that microorganisms belonging to the Chlamydiae class of the Verrucomicrobiota were significantly more frequent in samples from level 1 of the mine than in those of other levels. The mean Chlamydiae sequence content was 3.5% in samples from level 1, only 0.04% in level 4, and absent from level 3. The highest occurrence of the this taxon was observed in samples from the RD13 site, where Chlamydiae accounted for 10.07% of all ASVs in the 2017 season, and 12.28% in the 2019 season. Within the Chlamydiae class, sequences belonging to the *Simkaniaceae* family predominated, but sequences belonging to other families, such as Omnitrophaceae and Puniceicoccaceae, were also found at low frequency. This is an interesting observation because the detected microorganisms are not characteristic for a hypersaline environment. Bacteria belonging to the class Chlamydiae are obligate intracellular parasites. Therefore, it can be expected that the RD13 brine contained a large number of eukaryotic cells, probably in the form of Protists, which can be hosts for Chlamydiae bacteria. It is possible that the brines of level 1 were characterized by a lower salinity, which together with the Chlamydiae, was also manifested by a reduced presence of halophilic Archaea; however, this supposition was not confirmed by the chemical analysis of brines. Another explanation for the higher abundance of *Chlamydiae* on the first level of mine could be that they come from surface waters. Pawlikowska-Warych and Deptuła (2019) report the presence of bacteria from the *Simkaniaceae* family in surface waters in Poland; this would suggest that microorganisms from the surface water supplying brine outflows may reach the upper levels of the mine. Although the observation of *Chlamydiae* in such numbers in a hypersaline environment was unusual, similar observations had been made previously (Collingro et al. 2020).

Another interesting example was the case of the sample from site RA6, in which the phylum Patescibacteria was an important component of the microbiome: 21.6% of all ASVs in the sample from site RA6 belonged to this taxon, compared to 3.5%, which was average for all samples. This taxon includes microorganisms with highly reduced genomes (< 1Mbp). In the sample from site RA6, the dominant species within the Patescibacteria phylum was *Candidatus Falkowbacteria*, which constituted 18.6% of the microbiota composition of this site (Brown et al. 2015). These organisms were common in aquifers where conditions fluctuate between aerobic and anoxic (Anantharaman et al. 2016). They can also be related to the dissimilatory sulphur cycle in the environment (Anantharaman et al. 2018).

Some samples were found to harbour a large number of taxa not typically identified by 16S rRNA sequencing as extremophiles, such as Proteobacteria, Verrucomicrobiota, or Patescibacteria. This may suggest contamination of the Bochnia Salt Mine by a large number of halotolerants commonly identified in other environments. This is possible considering the flow of tourists, and surface water, into the mine; however, due to the very high salinity of the brines, exceeding 30% NaCl, it seems that survival in such conditions over a prolonged period would require the halotolerants to develop mechanisms similar to those of strict halophiles.

Furthermore, metagenomic data from shotgun sequencing found the mine microbiome to have slightly different characteristics. In contrast to the 16S rRNA gene sequencing, shotgun metagenomics found *Archaea* to be dominant in all samples. It was also noticed that fewer taxa were found at particular taxonomic levels compared to the 16S samples. Using shotgun sequencing, 16 unique MAGs were obtained with contamination less than 25% and completeness greater than 75%. Twelve out of 16 MAGs were assigned to *Archaea*, indicating that they were one of the most common microorganisms in the studied habitats.

It was only possible to assign a species to a single MAG: WSO4.4, which was assigned to the species *Halobacterium bonnevillei* (Myers and King 2020). Genomic sequence similarity analysis of the other MAGs indicated that they belong to currently unknown microorganism species. Interestingly, two of the received MAGs belong to the *Nanosalinaceae* family which is a part of the phylum Nanohaloarchaeota, which is part of DPANN superphylum. This taxon includes non-cultured microorganisms living in symbiosis with Halobacteria, characterized by small genomes and limited metabolic capabilities (Castelle et al. 2018; Xie et al. 2022). Due to the still incomplete characterization of microorganisms belonging to the Nanohaloarchaeota, the obtained MAGs may bring significant value to further research.

The results of the shotgun sequencing suggest that the high abundance of *Proteobacteria* indicated by the 16S rRNA results may not reflect the true presence of these bacteria in the environment: a methodological error may in fact favour the detection of this type of microorganism by 16S rRNA sequencing. Indeed, other researchers have also obtained high abundances of *Proteobacteria* in analyzed brines, example from the Karak Salt Mine or the graduation towers in the Ciechocinek city (Cycil et al. 2020; Kalwasińska et al. 2018).

The profiles of BGCs and AMP-encoding genes were also characterized in individual samples using AntiSMASH and DeepBGC. Only BGCs for terpenes were detected in all samples. The highest number of BGCs detected using both methods was noted in a sample from mine level 3; this was also the only sample in which BGCs for siderophore and thiopeptide production were detected. Siderophores are considered valuable products in agriculture, serving as plant growth–promoting factors or natural plant protection agents. They also have applications in medicine, as siderophore-antibiotic conjugates are effective against multi-drug-resistant pathogens, and can be used as imaging agents, and biosensors for metal ions and pathogens (Fan and Fang 2021; Ghosh et al. 2020). Thiopeptides are also useful in medicine where they can be applied as antibiotics (Wang et al. 2020). AMPs with haemolytic or antibacterial properties were detected in all samples, except for the one collected at level 6. Non-haemolytic, antibacterial properties are significant features supporting the use of AMP in clinical applications; however, even AMPs with haemolytic properties can find practical applications, such as nisin, which has been used in food preservation (Santos et al. 2018).

In conclusion, the presented study provides the first insight into the metagenome of the Bochnia Salt Mine. Our findings demonstrate that the microbiome of the mine is highly diverse and contains many potentially unknown species of microorganisms. It was also shown that the microorganisms inhabiting the mine contain numerous BGCs and AMP-encoding genes, which could be associated with valuable bioproducts.

## Supplementary Information

Below is the link to the electronic supplementary material.

Supplementary file1 (DOCX 354 KB)

## Data Availability

All sequencing data are publicly available from the National Institutes of Health under BioProject accession PRJNA848445. Other types of datasets used during the current study are available from the corresponding author upon reasonable request.
