# Supplementary material for: A first insight into the Polish Bochnia Salt Mine metagenome
Source: Environ Sci Pollut Res Int. 2023 Feb 13;30(17):49551–66. doi: 10.1007/s11356-023-25770-7 (PMC10104926; doi:10.1007/s11356-023-25770-7)
Supplement: Supplementary file 1 — Supplementary file1 (DOCX 354 KB) [file 11356_2023_25770_MOESM1_ESM.docx]

**Supplementary materials**

**Article Title:** First insight into the Polish Bochnia Salt Mine metagenome

**Appendix A: Tables**

Table S1. Quality statistics of *de novo* assembly of metagenomes and contigs binning

| Sample | # reads | # contigs > 1000 | # contigs > 5000 | N50 | Largest contig | Total len. (1000+) | Total len. (5000+) | # CDS | GC% | # Bins | # HQ Bins |
| --- | --- | --- | --- | --- | --- | --- | --- | --- | --- | --- | --- |
| POP27 | 21,103,558 | 23,169 | 2,755 | 4,037 | 167,367 | 68,499,871 | 29,983,054 | 48,938 | 61.7 | 17 | 5 |
| W1 | 20,587,500 | 12,477 | 506 | 2,097 | 225,954 | 25,265,906 | 4,484,516 | 14,004 | 52.0 | 11 | 2 |
| W81 | 31,251,888 | 14,443 | 2,199 | 5,098 | 117,083 | 49,647,332 | 25,033,518 | 39,350 | 58.7 | 21 | 7 |
| WSO4 | 31,396,622 | 25,733 | 2,551 | 3,514 | 100,580 | 71,844,617 | 28,839,859 | 49,587 | 58.8 | 23 | 6 |

Table S2. Quality statistics for bins

| Bin name | Completeness [%] | Contamination [%] | Strain heterogeneity [%] | Length [bp] | N50 [bp] |
| --- | --- | --- | --- | --- | --- |
| POP27.15 | 98,28 | 1,45 | 0 | 2,859,800 | 61,405 |
| POP27.16 | 88,87 | 2,76 | 26,09 | 3,224,161 | 12,393 |
| POP27.17 | 76,2 | 6,81 | 0 | 5,341,881 | 5,467 |
| W1.4 | 80,97 | 24,61 | 14,43 | 3,280,805 | 3,299 |
| W1.7 | 80,77 | 1,69 | 0 | 1,915,550 | 3,706 |
| W81.14 | 95,88 | 2,06 | 9,09 | 2,784,924 | 19,959 |
| W81.15 | 77,82 | 1,4 | 50 | 826,353 | 18,823 |
| W81.20 | 83 | 9,53 | 18,18 | 2,341,908 | 4,617 |
| W81.21 | 87,07 | 5,82 | 4,76 | 2,932,273 | 16,910 |
| W81.6 | 83,72 | 8,88 | 27,27 | 870,493 | 12,973 |
| W81.7 | 95,08 | 3,69 | 9,09 | 2,282,283 | 16,162 |
| W81.9 | 97,21 | 6,31 | 25,81 | 2,587,320 | 22,335 |
| WSO4.1 | 84,88 | 6,07 | 9,09 | 2,424,996 | 7,285 |
| WSO4.11 | 95,32 | 7,52 | 4 | 3,196,308 | 26,769 |
| WSO4.13 | 91,62 | 5,51 | 19,05 | 2,587,381 | 9,938 |
| WSO4.4 | 96,1 | 1,7 | 0 | 3,308,247 | 32,840 |

**Appendix B: Figures**


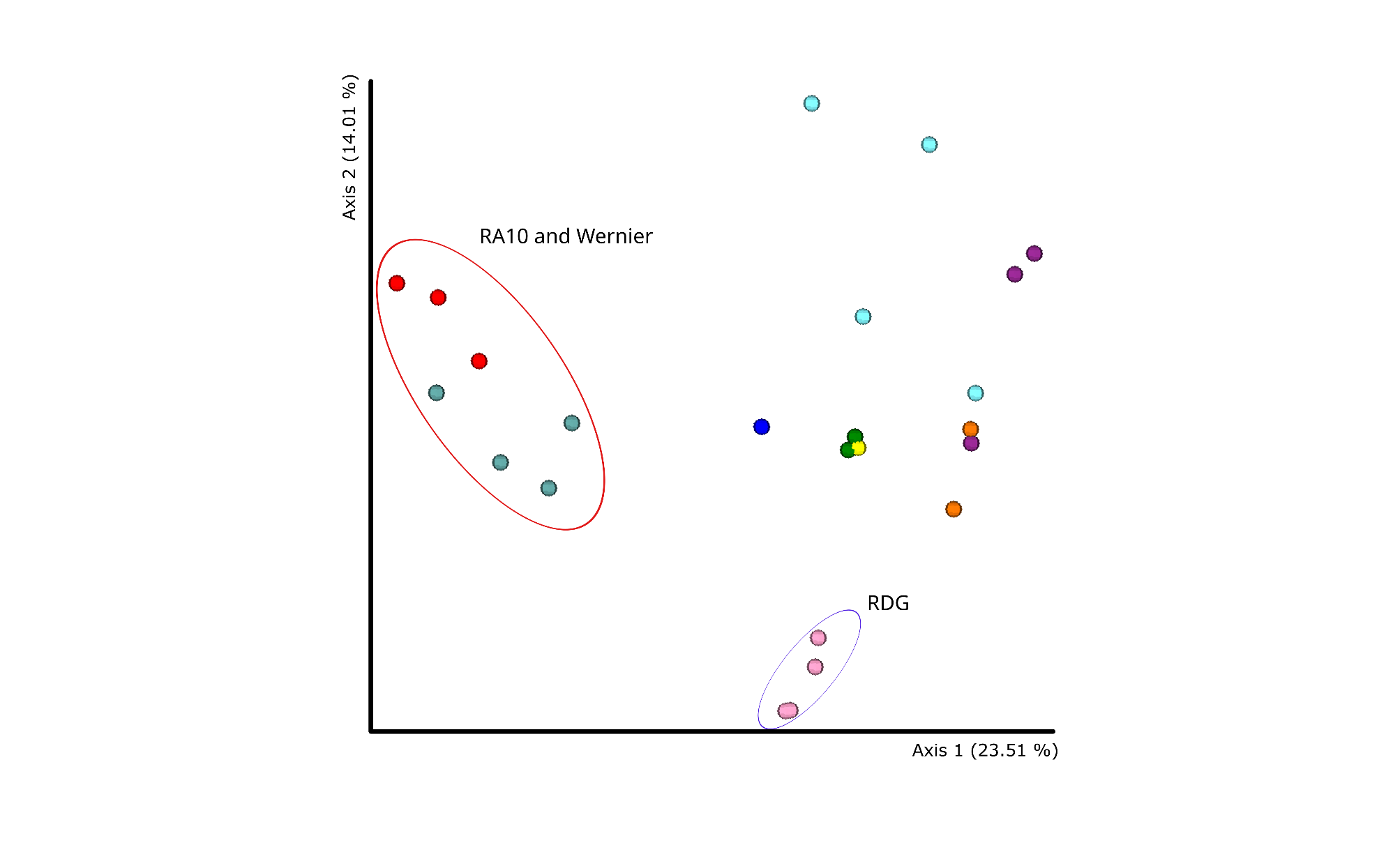


Figure S1. PCoA calculated on Bray-Curtis distance for samples segregated by location. Red – RA10 (level 4), cadet blue – Wernier (level 3), light blue – RDB (level 1), purple – RD13 (level 1), dark blue – RA3 (level 4), green – RD10 (level 1), yellow – RD3 (level 1), orange – RA6 (level 4), pink – RDG (level 1)


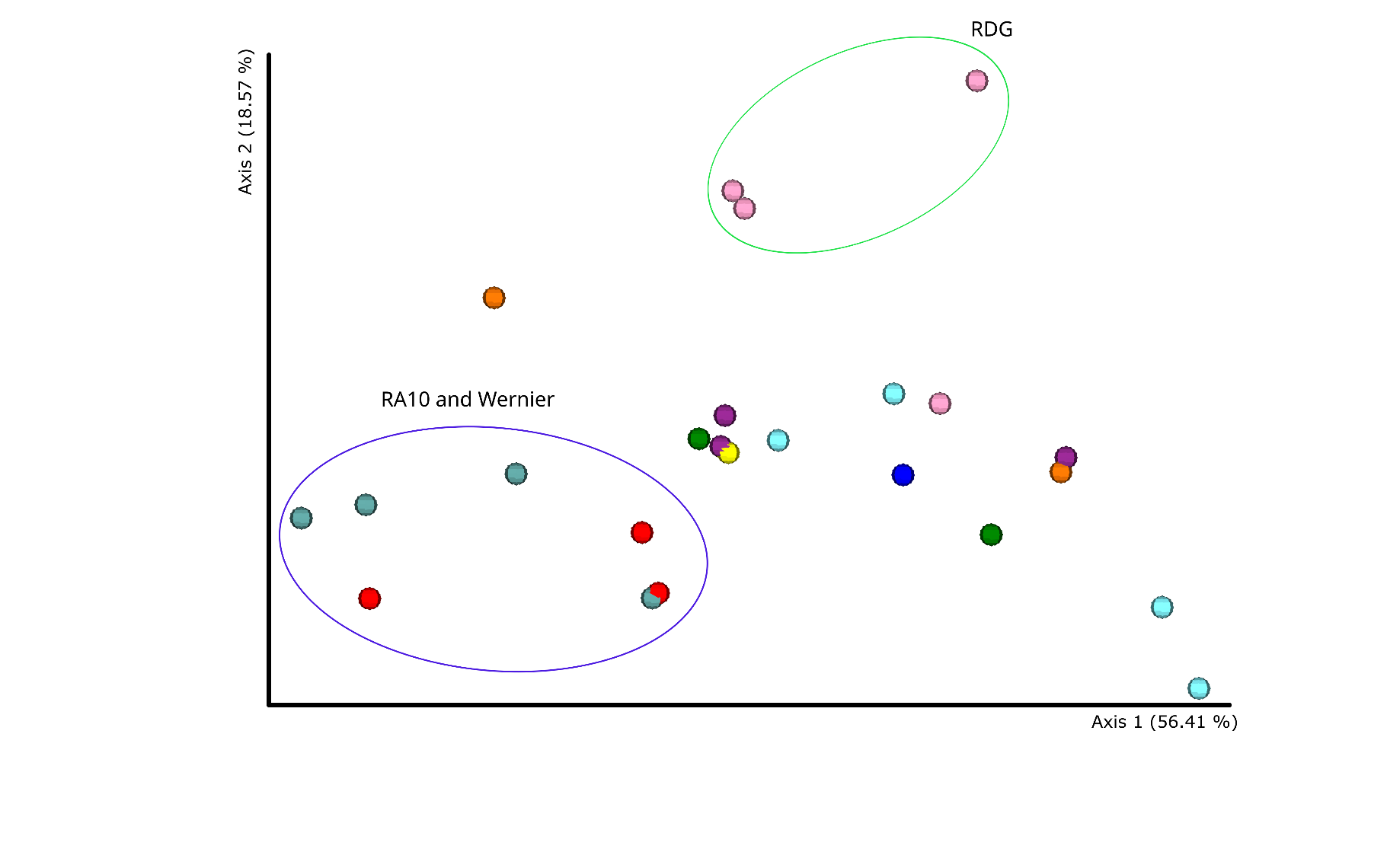


Figure S2. PCoA for Bray-Curtis distance calculated from metabolic pathway abundance for samples segregated by location. Red – RA10 (level 4), cadet blue – Wernier (level 3), light blue – RDB (level 1), purple – RD13 (level 1), dark blue – RA3 (level 4), green – RD10 (level 1), yellow – RD3 (level 1), orange – RA6 (level 4), pink – RDG (level 1)
